# Prevalence and Factors Associated With Vaping Cannabidiol Among US Adolescents

**DOI:** 10.1001/jamanetworkopen.2023.29167

**Published:** 2023-08-16

**Authors:** Hongying Daisy Dai, Roma Subramanian, Avina Mahroke, Ming Wang

**Affiliations:** 1 College of Public Health, University of Nebraska Medical Center, Omaha; 2 College of Osteopathic Medicine, Kansas City University, Kansas City, Missouri; 3 School of Communication, University of Nebraska at Omaha; 4 College of Journalism and Mass Communication, University of Nebraska-Lincoln

## Abstract

**Importance:**

e-Cigarette use and vaping marijuana (cannabis) are popular among US adolescents. Cannabidiol (CBD) is a compound found in the cannabis plant that has recently increased in use.

**Objectives:**

To examine the prevalence of and factors associated with youths vaping CBD.

**Design, Setting, and Participants:**

This cross-sectional study included a nationally representative sample of students from middle and high schools (typical age, 11-18 years) in the US from the 2022 National Youth Tobacco Survey, conducted from January to May 2022.

**Exposure:**

Demographic characteristics, harm perception of tobacco use, and vaping behaviors.

**Main Outcomes and Measures:**

The main outcomes were weighted prevalence and population estimates of ever and current (past 30-day) vaping of CBD overall and by e-cigarette use status. Multivariable logistic regression analyses were conducted to estimate the association of currently vaping CBD with demographic factors and e-cigarette and tobacco use behaviors stratified by current e-cigarette use status.

**Results:**

The study included 28 291 participants (51.1% male; mean [SD] age, 14.5 [2.0] years). Among 2448 current e-cigarette users, 21.3% (95% CI, 18.4%-24.1%) reported any past-month vaping of CBD and 6.3% (95% CI, 4.7%-7.8%) reported that they did not know whether they had vaped CBD. Hispanic e-cigarette users were more likely than their non-Hispanic White peers to report currently vaping CBD (adjusted odds ratio [AOR], 1.9; 95% CI, 1.3-2.8). Current e-cigarette users with higher frequency (≥20 days vs ≤5 days) and longer duration (2-3 years or >3 years vs <1 year) of use were more likely to report currently vaping CBD (frequency: AOR, 1.5 [95% CI, 1.1-1.9]; 2-3 years: AOR, 2.2 [95% CI, 1.2-3.9]; 3 years: AOR, 3.2 [95% CI, 1.7-6.1]). Among 25 091 noncurrent e-cigarette users, 1.2% (95% CI, 1.0%-1.5%) reported currently vaping CBD and 2.3% (95% CI, 2.1%-2.6%) reported that they did not know. High school students (vs middle school students; AOR, 4.2; 95% CI, 2.8-6.1) and gay or lesbian (AOR, 2.9; 95% CI, 1.6-5.4) or bisexual (AOR, 2.7; 95% CI, 1.8-4.0) (vs heterosexual) youths were more likely to report vaping CBD, while those who perceived tobacco as dangerous (vs not dangerous; AOR, 0.4; 95% CI, 0.3-0.6) had lower odds of reporting vaping CBD.

**Conclusions and Relevance:**

In this cross-sectional study of US middle and high school students, the prevalence of youths vaping CBD was high, particularly among e-cigarette users and Hispanic and sexual minority populations. The findings suggest that evidence-based educational campaigns, interventions, and public policy changes are needed to reduce the harmful health outcomes possible with vaping CBD among developing youths.

## Key Points

**Question:**

What is the prevalence of youths vaping cannabidiol (CBD), and what are the factors associated with this behavior?

**Findings:**

In this cross-sectional study of 28 291 adolescents, the prevalence of vaping CBD was high, particularly among current e-cigarette users and Hispanic and sexual minority populations. Viewing tobacco as harmful was associated with a reduced likelihood of vaping CBD.

**Meaning:**

The findings suggest that tighter CBD regulations to close existing loopholes and evidence-based educational campaigns and interventions are needed to reduce CBD vaping among youths.

## Teaser

This cross-sectional study assesses the prevalence of and factors associated with vaping cannabidiol among adolescents in the US overall and by e-cigarette use status.

## Introduction

Although Δ^9^-tetrahydrocannabinol (THC)–containing cannabis products remain illegal at the federal level in the US, the passage of the 2018 Agriculture Improvement Act, known as the 2018 Farm Bill,^1^ legalized the growth and sales of hemp, including cannabidiol (CBD) products containing hemp-derived CBD and up to 0.3% THC. While CBD products can be consumed in different modes, such as edibles (eg, gummies, candies), flowers, prerolled joints, concentrates (eg, potent extracts), and tinctures (eg, oil droppers),^2,3,4^ vaping CBD—that is, inhaling CBD via e-cigarettes and similar devices—has become one of the most popular modes of consuming CBD among young people.^3,4,5^ The perceived convenience and discreetness of vaping devices have contributed to the popularity of vaping cannabis products among youths.^6,7^ Notably, adolescent cannabis vaping has increased in recent years,^8^ with 4.2%, 10.3%, and 14.8% of US eighth-, tenth-, and twelfth-grade students reporting currently (past 30-day) vaping cannabis in 2022.^9^

Understanding the intersection of vaping CBD and e-cigarette use among adolescents has important implications for public health and regulatory actions. First, e-cigarettes have become the most commonly used tobacco product among youths since 2014 in the US,^10,11^ with 2.14 million US high school students (14.1%) and 380 000 middle school students (3.3%) reporting current (past 30-day) use in 2022.^12^ Identifying the prevalence of vaping CBD by e-cigarette use status can provide national estimates of the consequences of CBD for the health of the pediatric population. Second, youths vaping CBD is a public health concern given that CBD, nicotine, marijuana, and other compounds can be used interchangeably in some vaping devices, which can make it difficult to pinpoint the specific products responsible for the adverse health effects of vaping. This was evident in the e-cigarette or vaping product use–associated lung injury outbreak that occurred in 2019,^13^ which was linked to vitamin E acetate found in some THC-containing vaping products.^14^ Third, although CBD is the most popularized nonpsychoactive component of cannabis and does not cause a feeling of being high as THC does,^5^ the use of CBD raises several safety concerns, including potential harm to the liver and lungs and possible damage to the male reproductive system.^15^ In a recent study that compared the pulmonary effects of acute inhalation of vaporized CBD and nicotine, it was found that vaping CBD was associated with more severe lung damage than vaping nicotine.^16^ Gaining knowledge on subgroups of adolescents who engage in concurrent use of e-cigarettes and CBD vaping and those who exclusively vape CBD can provide valuable insights for developing targeted intervention approaches aimed at mitigating potential negative health consequences.

This study aimed to understand the characteristics of adolescents who vape CBD. Certain demographic factors that are associated with elevated use of adolescent e-cigarette use include being a sexual minority individual (vs heterosexual individual), an older individual (vs younger individual), and a non-Hispanic White individual (vs Black and Hispanic individuals).^12,17^ Understanding whether certain demographic populations are more likely to vape CBD can inform whether these products could potentially contribute to health disparities. Furthermore, harm perception of tobacco use, vaping frequency and duration, and e-cigarette devices used are important contributors to adolescent cannabis vaping.^8,18^ Assessing associations between vaping CBD with these tobacco use characteristics is vital as such information can elucidate how future public and regulatory policies addressing youth e-cigarette use should be designed in efforts to reduce the prevalence of youths vaping CBD. Thus, this study sought to address gaps in current literature on youths vaping CBD by (1) reporting prevalence and population estimates of ever and current (past 30-day) vaping of CBD among adolescents, (2) assessing associations of demographic factors and tobacco use behaviors with vaping CBD, and (3) providing comparisons of prevalence of and factors associated with youths vaping CBD between e-cigarette users and nonusers.

## Methods

### Study Sample

This cross-sectional study used data from the National Youth Tobacco Survey (NYTS), which annually conducts a school-based survey to gather a representative sample of US middle school (grades 6-8) and high school (grades 9-12) students (ages 11-18 years). The 2022 NYTS used a stratified, 3-stage cluster sampling procedure and collected data from January to May 2022. The survey was completed by 28 291 students from 341 schools, resulting in a school participation rate of 59.4%, a student response rate of 76.1%, and an overall response rate of 45.2%.^12^ In 2022, the survey was administered online to allow eligible students to complete the survey at home, at school, or somewhere else. A detailed description of the 2022 NYTS survey can be found on the NYTS website.^19^ NYTS study participation required parental consent and student assent. Given the use of public data with deidentified information, this study was treated as non–human participant research and was exempted from University of Nebraska Medical Center institutional review board approval. This study followed the Strengthening the Reporting of Observational Studies in Epidemiology (STROBE) reporting guideline for cohort studies.^20^

### Measures

#### Vaping CBD

Participants were asked, “Have you ever vaped any of the following substances (even once)? CBD or CBD oils,” with response options of yes, no, and don’t know. Those who responded yes or don’t know were further asked, “Have you vaped any of the following substances during the past 30 days? CBD or CBD oils,” with response options of yes, no, and don’t know. We included all 3 statuses (yes, no and don’t know) for ever and current (past 30-day) vaping of CBD.

#### e-Cigarette Use and Other Tobacco Use Status

Ever e-cigarette users were classified as those who responded yes to the question, “Have you ever used an e-cigarette, even once or twice?” Students who reported having used e-cigarettes 1 day or more in the past 30 days were classified as current e-cigarette users.^21^ Similarly, we created ever and current (≥1 day in the past 30 days) use status of any other tobacco product, including cigarettes, cigars (cigars, little cigars, and cigarillos), smokeless tobacco (chewing tobacco, snuff, dip, snus, and dissolvable tobacco), hookahs, pipe tobacco, bidis, and heated tobacco product.^22^

#### Vaping and Tobacco Use Behaviors

We categorized current e-cigarette users as occasional users (≤5 days), moderate users (6-19 days), and frequent users (≥20 days) based on the frequency of e-cigarette use in the past 30 days.^21^ Given the difference between the age when respondents first used an e-cigarette and their current age, we derived vaping duration as 4 mutually exclusive groups: less than 1 year, 1 year, 2 to 3 years, and more than 3 years.^22^ Vaping product used in the past 30 days was determined by the question, “Which of the following best describes the type of e-cigarette you have used in the past 30 days?” Response options were “a disposable e-cigarette,” “an e-cigarette that uses prefilled pods or cartridges (eg, JUUL),” “an e-cigarette with a tank that you refill with liquids,” “a mod system (an e-cigarette that can be customized by the user with their own combination of batteries or other),” and “I don’t know the type.” The perceived danger of tobacco use was measured by the question, “How strongly do you agree with the statement ‘All tobacco products are dangerous’?” We classified respondents into perceiving tobacco use as dangerous (those who responded strongly agree or agree) or not dangerous (those who responded disagree or strongly disagree).^23^

#### Other Covariates

Demographic variables included sex (female or male), race and ethnicity (Hispanic, non-Hispanic Black, non-Hispanic White, or non-Hispanic other [American Indian or Alaska Native, Asian, and Native Hawaiian or Other Pacific Islander]), school level (middle or high school), sexual identity (bisexual, gay or lesbian, heterosexual, and not sure), and speaking a language other than English at home (yes or no). Self-reported race and ethnicity were included as social constructs instead of biological or generic categories.

### Statistical Analysis

After descriptive analyses, we estimated sample sizes, population estimates, and weighted prevalence of ever and current vaping of CBD overall and stratified by e-cigarette use status. In the multivariable analyses, separate logistic regressions were conducted to estimate the association of current vaping of CBD with demographic factors and e-cigarette and tobacco use behaviors stratified by current e-cigarette use status (no and yes). The supplemental analyses also included multivariable analyses of factors associated with ever vaping CBD.

Sampling weights, survey stratum, and primary sampling units were included in all analyses to account for the complex survey design and adjust for nonresponse. Missing covariate data were managed with multiple imputations using 20 multiply-imputed data sets.^24^ Adjusted odds ratios (AOR) and *P* values were reported in the multivariable analyses. Statistical analyses were performed using SAS, version 9.4 (SAS Institute Inc), and 2-sided *P* values <.05 were considered statistically significant.

## Results

The study sample included 28 291 youths (mean [SD] age, 14.5 [2.0] years). Of these youths, 48.9% (95% CI, 47.9%-49.9%) were female; 51.1% (95% CI, 50.1%-52.1%), male; 43.8% (95% CI, 37.5%-50.1%), middle school students; 56.2% (95% CI, 49.9%-62.5%), high school students; 26.9% (95% CI, 23.0%-30.8%), Hispanic; 12.4% (95% CI, 10.3%-14.4%), non-Hispanic Black; 54.1% (95% CI, 48.9%-59.3%), non-Hispanic White; and 6.6% (95% CI, 3.6%-9.6%), non-Hispanic other race and ethnicity (Table 1). Also, 11.8% (95%CI, 11.1%-12.5%) identified as bisexual; 4.5% (95% CI, 4.1%-5.0%), as gay or lesbian; 73.8% (95% CI, 72.6%-75.0%), as heterosexual; and 9.9% (95% CI, 9.1%-10.7%), as unsure. Of the participants, 30.5% (95% CI, 26.0%-34.9%) reported speaking a language other than English at home.

**Table 1.** Characteristics of the 2022 National Youth Tobacco Survey Sample

Characteristic	Participants	
	No.	Weighted % (95% CI)
Overall	28 291	100
Sex		
Female	13 692	48.9 (47.9-49.9)
Male	14 375	51.1 (50.1-52.1)
Grade		
Middle school	12 041	43.8 (37.5-50.1)
High school	16 118	56.2 (49.9-62.5)
Race and ethnicity		
Hispanic	7458	26.9 (23.0-30.8)
Non-Hispanic Black	3383	12.4 (10.3-14.4)
Non-Hispanic White	13 501	54.1 (48.9-59.3)
Non-Hispanic other^a^	3171	6.6 (3.6-9.6)
Sexual identity		
Bisexual	3002	11.8 (11.1-12.5)
Gay or lesbian	1146	4.5 (4.1-5.0)
Heterosexual	18 740	73.8 (72.6-75)
Unsure	2547	9.9 (9.1-10.7)
Language other than English at home		
No	17 595	69.5 (65.1-74.0)
Yes	8036	30.5 (26.0-34.9)

^a^ Other races included American Indian or Alaska Native, Asian, and Native Hawaiian or Other Pacific Islander.

Table 2 presents the prevalence and population estimates of ever and current vaping of CBD overall and stratified by e-cigarette use status. Overall, an estimated 1 869 000 participants (7.0%; 95% CI, 6.0%-8.0%) reported ever vaping CBD and 1 568 000 (5.9%; 95% CI, 5.4%-6.4%) reported that they did not know whether they had ever vaped CBD; an estimated 796 000 participants (3.0%; 95% CI, 2.5%-3.5%) reported current (past 30-day) vaping of CBD, and 735 000 (2.7%; 95% CI, 2.4%-3.1%) reported that they did not know about currently vaping CBD. Of the 796 000 students currently vaping CBD, 62.1% reported current (past 30-day) e-cigarette use. Among those who reported they did not know about ever vaping CBD, 989 000 were never e-cigarette users and 570 000 were ever e-cigarette users. Vaping CBD was more pronounced among e-cigarette users than among nonusers. For instance, the prevalence of current vaping of CBD was 21.3% (95% CI, 18.4%-24.1%) among current e-cigarette users compared with 1.2% (95% CI, 1.0%-1.5%) among noncurrent e-cigarette users, while 6.3% (95% CI, 4.7%-7.8%) of current e-cigarette users reported they did not know about currently vaping CBD compared with 2.3% (95% CI, 2.1%-2.6%) of noncurrent e-cigarette users.

**Table 2.** Prevalence and Population Estimates of Ever and Current Vaping of CBD in 2022

e-Cigarette use	Overall, unweighted No.	Vaped CBD			Do not know about vaping CBD		
		Unweighted No.	Weighted No.^a^	Weighted % (95% CI)^a^	Unweighted No.	Weighted No.^a^	Weighted % (95% CI)^a^
**Ever vaped CBD**							
Overall	27 412	1964	1 869 000	7.0 (6.0-8.0)	1696	1 568 000	5.9 (5.4-6.4)
Never e-cigarette user	21 977	546	457 000	2.1 (1.7-2.5)	1057	989 000	4.6 (4.1-5.2)
Ever e-cigarette user	5435	1418	1 412 000	27.4 (25.2-29.7)	634	570 000	11.1 (9.5-12.6)
**Current vaping of CBD**							
Overall	27 539	841	796 000	3.0 (2.5-3.5)	797	735 000	2.7 (2.4-3.1)
Noncurrent e-cigarette user	25 091	333	298 000	1.2 (1.0-1.5)	600	572 000	2.3 (2.1-2.6)
Current e-cigarette user	2448	498	494 000	21.3 (18.4-24.1)	184	146 000	6.3 (4.7-7.8)

Abbreviation: CBD, cannabidiol.

^a^ Sampling weights, survey stratum, and primary sampling units were applied to account for the complex survey design and estimate the population totals, which were rounded to 1000.

As shown in Table 3, among current e-cigarette users (n = 2448), Hispanic individuals were more likely than their non-Hispanic White peers to report currently vaping CBD (25.9% [95% CI, 20.4%-31.4%] vs 18.7% [95% CI, 15.0%-22.4%]; AOR, 1.9 [95% CI, 1.3-2.8]). Current e-cigarette users with a higher frequency of use (≥20 days, 27.6% [95% CI, 23.4%-31.8%] vs ≤5 days, 15.2% [95% CI, 11.9%-18.5%]; AOR, 1.5 [95% CI, 1.1-1.9]) and a longer duration of use (2-3 years vs <1 year: AOR, 2.2 [95% CI, 1.2-3.9]; >3 years vs 1 year: AOR, 3.2 [95% CI, 1.7-6.1]) were also more likely to report currently vaping CBD. Current e-cigarette users who also reported use of other tobacco products also had higher odds of reporting currently vaping CBD (AOR, 2.5; 95% CI, 1.7-3.8). Furthermore, those who perceived tobacco use as dangerous (vs not dangerous) or those who did not know what was in their vaping devices (vs those reporting use of a disposable e-cigarette) were more likely to report that they did not know about currently vaping CBD.

**Table 3.** Factors Associated With Current Vaping of CBD Among Current e-Cigarette Users

Factor	Weighted prevalence of current vaping of CBD, % (95% CI)		Current vaping of CBD^a^			
			Yes vs no		Do not know vs no	
	Yes	Do not know	AOR	*P* value	AOR	*P* value
Overall	21.3 (18.4-24.1)	6.3 (4.7-7.8)	NA	NA	NA	NA
Sex						
Female	20.9 (17.4-24.4)	6.1 (4.3-7.9)	1.1 (0.7-1.7)	.61	0.8 (0.5-1.4)	.50
Male	21.6 (16.3-26.9)	6.5 (4.3-8.8)	1 [Reference]	NA	1 [Reference]	NA
Grade						
Middle school	15.4 (9.1-21.6)	10.1 (6.6-13.6)	1 [Reference]	NA	1 [Reference]	NA
High school	22.0 (18.9-25.0)	5.6 (3.9-7.4)	1.3 (0.8-2.2)	.31	0.7 (0.4-1.2)	.20
Race and ethnicity						
Hispanic	25.9 (20.4-31.4)	7.3 (4.3-10.3)	1.9 (1.3-2.8)	<.001	1.1 (0.6-2.0)	.83
Non-Hispanic Black	26.1 (16.9-35.2)	8.1 (3.6-12.6)	1.8 (1.0-3.5)	.06	1.3 (0.5-2.9)	.58
Non-Hispanic White	18.7 (15.0-22.4)	5.6 (3.3-7.8)	1 [Reference]	NA	1 [Reference]	NA
Non-Hispanic other^b^	19.0 (7.0-30.9)	7.3 (2.3-12.3)	1.2 (0.5-2.7)	.68	1.1 (0.4-3.2)	.88
Sexual identity						
Bisexual	22.3 (16.0-28.6)	8.1 (4.3-12)	1.3 (0.8-2.1)	.29	1.6 (0.8-3.1)	.19
Gay or lesbian	28.6 (17.1-40.1)	5.9 (0.0-12.7)	1.5 (0.9-2.5)	.15	1.2 (0.5-3.2)	.66
Heterosexual	18.2 (14.9-21.6)	5.2 (3.2-7.3)	1 [Reference]	NA	1 [Reference]	NA
Unsure	27.4 (13.4-41.5)	7.8 (0-16.6)	1.6 (0.7-3.6)	.26	1.3 (0.3-5.1)	.69
Language other than English at home						
No	20.8 (17.5-24.1)	4.9 (2.9-6.9)	1 [Reference]	NA	1 [Reference]	NA
Yes	18.1 (13.0-23.2)	9.3 (5.4-13.1)	0.8 (0.5-1.3)	.34	1.5 (0.8-2.9)	.23
Perceived tobacco danger						
No	25.8 (20.2-31.4)	8.3 (5.3-11.4)	1 [Reference]	NA	1 [Reference]	NA
Yes	19.8 (16.5-23.1)	5.0 (3.3-6.7)	0.9 (0.6-1.3)	.51	0.6 (0.32-0.98)	.04
Frequency of e-cigarette use^c^						
Occasional	15.2 (11.9-18.5)	9.2 (6.7-11.6)	1 [Reference]	NA	1 [Reference]	NA
Moderate	19.9 (13.2-26.7)	4.4 (1.8-7.0)	1.1 (0.7-1.8)	.71	0.5 (0.24-0.98)	.04
Frequent	27.6 (23.4-31.8)	4.2 (1.7-6.7)	1.5 (1.1-1.9)	.01	0.6 (0.3-1.2)	.16
Vaping duration, y						
<1	9.3 (5.4-13.3)	8.9 (4.6-13.2)	1 [Reference]	NA	1 [Reference]	NA
1	17.3 (12.6-22.1)	6.7 (3.6-9.9)	2.0 (1.0-4.0)	.05	0.9 (0.4-2.0)	.82
2-3	20.9 (15.7-26.1)	5.9 (3.4-8.4)	2.2 (1.2-3.9)	.01	1.1 (0.5-2.6)	.81
>3	31.6 (26.8-36.4)	5.1 (2.3-7.9)	3.2 (1.7-6.1)	<.001	1.2 (0.5-2.8)	.70
Type of e-cigarette used in the past 30 d						
Disposable e-cigarette	21.4 (18.4-24.3)	5.8 (3.6-7.9)	1 [Reference]	NA	1 [Reference]	NA
e-Cigarette that uses prefilled pods or cartridges	22.5 (16.6-28.4)	4.1 (2.2-5.9)	0.9 (0.6-1.3)	.54	0.7 (0.4-1.3)	.23
e-Cigarette with a tank that is refilled with liquids	29.5 (18.4-40.6)	7.6 (1.2-14)	1.4 (0.8-2.5)	.23	1.2 (0.4-3.7)	.70
Do not know	13.4 (7.6-19.1)	12.5 (7.9-17.1)	0.7 (0.4-1.2)	.21	1.7 (1.0-2.9)	.046
Current use of other tobacco products						
No	15.4 (12.0-18.7)	6.7 (4.9-8.6)	1 [Reference]	NA	1 [Reference]	NA
Yes	34.3 (27.5-41.1)	5.2 (3.1-7.3)	2.5 (1.7-3.8)	<.001	1.0 (0.6-1.7)	.90

Abbreviations: AOR, adjusted odds ratio; CBD, cannabidiol; NA, not applicable.

^a^ Multinomial logistic regression model included current vaping of CBD (no as the reference group) as the outcome variable, with all variables in this table as simultaneous regressors. Missing covariate data were managed with multiple imputations using 20 multiply-imputed data sets.

^b^ Included American Indian or Alaska Native, Asian, and Native Hawaiian or other Pacific Islander.

^c^ Occasional was defined as 5 days or less; moderate, 6 to 19 days; and frequent, 20 days or more.

Table 4 presents the prevalence of and factors associated with currently vaping CBD among noncurrent e-cigarette users (n = 25 091). High school students vs middle school students (1.9% [95% CI, 1.5%-2.2%] vs 0.5% [95% CI, 0.3%-0.6%]; AOR, 4.2 [95% CI, 2.8-6.1]) and those who identified as bisexual or gay or lesbian vs heterosexual (bisexual: AOR, 2.7 [95% CI, 1.8-4.0]; gay or lesbian: AOR, 2.9 [95% CI, 1.6-5.4]) had higher odds of reporting currently vaping CBD. Noncurrent e-cigarette users who perceived tobacco use to be dangerous vs not dangerous (1.0% [95% CI, 0.7%-1.2%] vs 2.6% [95% CI, 1.7%-3.5%]; AOR, 0.4 [95% CI, 0.3-0.6]) were less likely to report currently vaping CBD.

**Table 4.** Factors Associated With Current Vaping of CBD Among Noncurrent e-Cigarette Users

Factor	Weighted prevalence of current vaping of CBD (95% CI)		Current vaping of CBD^a^			
			Yes vs no		Do not know vs no	
	Yes	Do not know	AOR	*P* value	AOR	*P* value
Overall	1.2 (1-1.5)	2.3 (2.1-2.6)	NA	NA	NA	NA
Sex						
Female	1.4 (1.0-1.7)	2.0 (1.6-2.4)	1.0 (0.7-1.4)	.85	0.7 (0.5-0.9)	.01
Male	1.1 (0.8-1.4)	2.7 (2.3-3.1)	1 [Reference]	NA	1 [Reference]	NA
Grade						
Middle school	0.5 (0.3-0.6)	2.3 (2.0-2.6)	1 [Reference]	NA	1 [Reference]	NA
High school	1.9 (1.5-2.2)	2.4 (1.9-2.8)	4.2 (2.8-6.1)	<.001	1.1 (0.9-1.3)	.53
Race/ethnicity						
Hispanic	1.5 (1.0-2.1)	3.1 (2.4-3.8)	1.2 (0.7-1.9)	.57	1.5 (0.9-2.3)	.09
Non-Hispanic Black	1.3 (0.9-1.8)	3.4 (2.3-4.5)	1.0 (0.7-1.5)	.82	1.8 (1.1-2.8)	.01
Non-Hispanic White	1.1 (0.8-1.4)	1.7 (1.3-2.2)	1 [Reference]	NA	1 [Reference]	NA
Non-Hispanic other^b^	0.7 (0.0-1.3)	2.0 (1.2-2.8)	0.6 (0.2-1.6)	.28	1.0 (0.6-1.7)	.93
Sexual identity						
Bisexual	2.4 (1.6-3.2)	2.2 (1.3-3.1)	2.7 (1.8-4.0)	<.001	1.3 (0.8-1.9)	.29
Gay or lesbian	2.9 (1.0-4.7)	2.5 (0.9-4.1)	2.9 (1.6-5.4)	<.001	1.5 (0.9-2.5)	.15
Heterosexual	0.9 (0.6-1.1)	1.9 (1.7-2.2)	1 [Reference]	NA	1 [Reference]	NA
Unsure	0.7 (0.2-1.3)	4.0 (2.9-5.1)	1.0 (0.5-2.0)	.96	2.0 (1.5-2.6)	<.001
Language other than English at home						
No	1.0 (0.7-1.3)	1.7 (1.5-2.0)	1 [Reference]	NA	1 [Reference]	NA
Yes	1.2 (0.8-1.7)	3.0 (2.3-3.7)	1.3 (0.8-2.1)	.34	1.4 (0.9-2.0)	.09
Perceived tobacco danger						
No	2.6 (1.7-3.5)	6.1 (4.6-7.6)	1 [Reference]	NA	1 [Reference]	NA
Yes	1.0 (0.7-1.2)	1.7 (1.4-1.9)	0.4 (0.3-0.6)	<.001	0.3 (0.2-0.4)	<.001

Abbreviations: AOR, adjusted odds ratio; CBD, cannabidiol; NA, not applicable.

^a^ Multinomial logistic regression model included current vaping of CBD (no as the reference group) as the outcome variable, with all variables in this table as simultaneous regressors. Missing covariate data were managed with multiple imputation using 20 multiply-imputed data sets.

^b^ Included American Indian or Alaska Native, Asian, and Native Hawaiian or other Pacific Islander.

Among noncurrent e-cigarette users, female vs male participants (2.0% [95% CI, 1.6%-2.4%] vs 2.7% [95% CI, 2.3%-3.1%]; AOR, 0.7 [95% CI, 0.5-0.9]), non-Hispanic Black vs non-Hispanic White participants (3.4% [95% CI, 2.3%-4.5%] vs 1.7% [95% CI, 1.3%-2.2%]; AOR, 1.8 [95% CI, 1.1-2.8]), and those unsure about their sexual identity vs heterosexual participants (4.0% [95% CI, 2.9%-5.1%] vs 1.9% [95% CI, 1.7%-2.2%]; AOR, 2.0 [95% CI, 1.5-2.6]) had higher odds of reporting that they did not know about currently vaping CBD. Those who perceived tobacco to be dangerous vs not dangerous (1.7% [95% CI, 1.4%-1.9%] vs 6.1% [95% CI, 4.6%-7.6%]; AOR, 0.3 [95% CI, 0.2-0.4]) were less likely to report that they did not know about currently vaping CBD.

The prevalence of ever vaping CBD by factors associated with vaping and multivariable results of factors associated with ever vaping CBD among never and ever e-cigarette users are presented in eTables 1 and 2 in Supplement 1, respectively. The results were generally consistent with those for current vaping of CBD.

## Discussion

A growing number of studies have assessed the use of CBD among US adults, including vaping CBD.^3,4,5^ For instance, a 2020 study conducted in 6 US cities estimated that about 32.0% of individuals aged 18 to 34 years reported current use of CBD, and 40.7% of them also reported vaporizer or vape pen as the most common way to use CBD.^4^ To our knowledge, this study is the first to examine vaping CBD among adolescents and provides population estimates by analyzing the nationally representative NYTS data set. In 2022, 1 869 000 US middle and high school students reported ever vaping CBD and 796 000 reported vaping CBD in the past 30 days.

The intersection of vaping CBD and vaping e-cigarettes creates a new challenge to regulate vaping products. Our study found that the prevalence of vaping CBD was particularly pronounced among e-cigarette users. The prevalence of current vaping of CBD was higher among current e-cigarette users than noncurrent e-cigarette users (21.3% vs 1.2%). Furthermore, among those who reported currently vaping CBD, 62.1% also reported e-cigarette use in the past 30 days. While e-cigarette manufacturers are required to submit premarket tobacco applications to the Food and Drug Administration (FDA) before marketing and distributing their products in the US, the same requirement does not extend to CBD. Currently, CBD products are loosely regulated as they are classified as dietary supplements on the market.^25^ Due to the lack of regulation, many CBD producers and retailers have marketed CBD with brightly colored packaging and advertisements, a variety of flavors (eg, sweet and fruity) in liquid, and products like chocolates, gummies, cookies, and brownies to attract young people.^2,26^ Furthermore, CBD is also sold in various settings, including pharmacies, vape shops, online, and grocery stores.^4,5,25^ This is particularly noteworthy as CBD products are accessible in several states where legal cannabis is not yet available, and there is no standard minimum purchasing age for these products.^2^ In January 2023, the FDA determined that the current regulatory frameworks for foods and supplements are inadequate for CBD products.^27^ As a result, prompt regulatory measures are necessary to address the existing loophole in the marketing and distribution of CBD products, which can lead to increased adolescent use of CBD through vaping and coadministration with other substances like e-cigarettes.

This study also identified important factors associated with youths vaping CBD. Among current e-cigarette users, Hispanic students had higher odds of reporting current CBD use than their non-Hispanic White peers. While the prevalence of e-cigarette use was generally lower among Hispanic adolescents compared with their White counterparts in a national sample (eg, 6.0% vs 9.6%) in the 2021 NYTS study,^28^ other studies have indicated a higher prevalence of cannabis vaping among Hispanic students compared with other racial and ethnic groups.^8,18,29^ For instance, Dai^8^ estimated that 18.4% of Hispanic middle and high school students reported cannabis vaping in 2018 compared with 14.5% of their non-Hispanic counterparts. The higher prevalence of cannabis vaping and CBD vaping among Hispanic youths compared with White youths may be associated with potential lower harm perception of cannabis use and lower media literacy.^30,31^ Among noncurrent e-cigarette users, this study also identified a higher prevalence of current CBD vaping among gay or lesbian and bisexual students than their heterosexual counterparts. Sexual minority adolescents may experience elevated levels of stress due to social stigma and marginalization^32,33^ and report higher e-cigarette use, nicotine dependence, and co-use of other tobacco products.^17,34^ The elevated risk of vaping CBD among these subpopulations, such as sexual minority groups and Hispanic individuals, is a concern given that they typically experience a disproportionate burden of adverse health outcomes.^35,36^ In addition to demographic factors, this study found positive associations of vaping frequency and duration with current vaping of CBD and also identified the perceived tobacco danger to be a protective factor associated with vaping CBD, especially among non–e-cigarette users. The FDA launched The Real Cost E-Cigarette Prevention Campaign in 2018^37^ to educate young individuals about the potential health hazards in vaping nicotine. Continuous education of youths about the harms of vaping, including the use of CBD, is warranted to advance health equity.

A substantial number of US adolescents also reported that they were unsure whether they had vaped CBD, including an estimated 989 000 never e-cigarette users and 570 000 ever e-cigarette users. Notably, among never e-cigarette users, the prevalence of those who reported that they did not know about ever vaping CBD was higher among Hispanic and non-Hispanic Black participants (vs non-Hispanic White participants) and among those unsure about their sexual identity (vs heterosexual peers). One possible reason why adolescents are unsure about vaping CBD is the lack of consistent quality-assurance standards. A substantial number of CBD items have ambiguous labeling or discrepancies between the listed components and the actual composition of the product.^38^ A prior study reported that only 3 of the 20 popular CBD products available in the market matched their label claims.^39^ This could result in mislabeling of current CBD products in the market and may also lead to youth misidentifying them as other vaping products. In addition, CBD is often advertised as a food and health supplement.^25^ Despite the lack of effective evidence, some products are marketed with explicit health claims of the therapeutic benefits of CBD use, including in the treatment of anxiety, inflammation, pain, and stress.^26^ Therefore, adolescents might view them as dietary supplements and thus may be unsure about the types of substances present in their vaping products.

### Limitations

This study has some limitations. First, self-reported vaping of CBD is subject to recall and social desirability biases. However, the self-reported behaviors related to substance use among adolescents have demonstrated high test-retest reliability.^40^ Second, the 2022 NYTS only asked for ever and past 30-day vaping of CBD without additional measures about the frequency and dosage of vaping CBD; thus, we were unable to provide nuanced details of behaviors of youths vaping CBD. Third, this study provided evidence on youths vaping CBD, one of the primary sources of CBD use. However, the NYTS lacks questions to assess the full picture of other CBD use sources (eg, edibles, oil extracts, and flowers). Overall CBD use may be higher than the reported values specifically for CBD vaping in this study, and future studies should assess youth CBD use across different product sources. Fourth, the cross-sectional NYTS data preclude causal inference.

## Conclusion

In this cross-sectional study of a nationally representative sample of US middle and high school students, the prevalence of youths vaping CBD was high, with Hispanic and sexual minority populations having particularly high prevalence. The findings call for evidence-based educational campaigns and interventions to reduce CBD vaping among youths. Tighter regulations also may be necessary to address the concurrent use of CBD vaping and e-cigarettes and to close existing loopholes that undermine the health of young people. Enforcement and implementation of regulatory actions such as age restrictions and product labeling requirements appear to be needed to reduce the prevalence of adolescent vaping of CBD.

## Notes

Supplement 1. **eTable 1.** Factors Correlated With Ever Vaping CBD Among Never E-cigarette Users **eTable 2.** Factors Correlated With Ever Vaping CBD Among Ever E-cigarette Users

Supplement 2.
Data Sharing Statement
