# Supplementary material for: Prevalence and Factors Associated With Vaping Cannabidiol Among US Adolescents
Source: JAMA Netw Open. 2023 Aug 16;6(8):e2329167. doi: 10.1001/jamanetworkopen.2023.29167 (PMC10433080; doi:10.1001/jamanetworkopen.2023.29167)
Supplement: Supplement 1. — eTable 1. Factors Correlated With Ever Vaping CBD Among Never E-cigarette Users eTable 2. Factors Correlated With Ever Vaping CBD Among Ever E-cigarette Users [file jamanetwopen-e2329167-s001.pdf]

Supplemental Online Content

Dai HD, Subramanian R, Mahroke A, Wang M. Prevalence and factors associated with vaping cannabidiol among US adolescents. *JAMA Netw Open.* 2023;6(8):e2329167. doi:10.1001/jamanetworkopen.2023.29167

eTable 1. Factors Correlated With Ever Vaping CBD Among Never E-cigarette Users

eTable 2. Factors Correlated With Ever Vaping CBD Among Ever E-cigarette Users

This supplemental material has been provided by the authors to give readers additional information about their work.

© 2023 Dai HD et al. *JAMA Network Open.*

eTable 1. Factors Correlated with Ever Vaping CBD among Never E-cigarette Users

				Ever Vaping CBD			
		Prevalence of Ever Vaping CBD		Yes vs. No		I don't know vs. No	
		Yes	Don't Know	AOR[a]	P-value[a]	AOR[a]	P-value[a]
		2.1 (1.7 to 2.5)	4.6 (4.1 to 5.2)				
Sex							
	Male	1.8 (1.4 to 2.2)	5.0 (4.4 to 5.6)	Ref	Ref	Ref	Ref
	Female	2.4 (1.9 to 3.0)	4.1 (3.4 to 4.8)	1.2 (0.9 to 1.5)	0.19	0.8 (0.7 to 0.9)	0.01
Grade							
	Middle School	0.9 (0.7 to 1.2)	5.4 (4.7 to 6.1)	Ref	Ref	Ref	Ref
	High School	3.3 (2.7 to 3.9)	3.8 (3.1 to 4.5)	3.6 (2.6 to 4.8)	<.0001	0.7 (0.6 to 0.9)	0.001
Race / Ethnicity							
	NH-white	1.7 (1.3 to 2.2)	3.6 (3.0 to 4.2)	Ref	Ref	Ref	Ref
	NH-black	3.1 (2.2 to 4.0)	5.1 (4.1 to 6.2)	1.6 (1.1 to 2.4)	0.01	1.4 (1.1 to 1.8)	0.01
	Hispanic	2.9 (2.1 to 3.7)	6 (4.8 to 7.3)	1.8 (1.1 to 2.8)	0.01	1.4 (1.1 to 1.9)	0.01
	Others	1.1 (0.4 to 1.8)	4.6 (3.3 to 5.9)	0.7 (0.3 to 1.7)	0.47	1.1 (0.8 to 1.5)	0.60
Sexual Minority							
	Heterosexual	1.7 (1.3 to 2.1)	4.0 (3.5 to 4.5)	Ref	Ref	Ref	Ref
	Gay/Lesbian	3.1 (1.3 to 4.9)	3.8 (2.4 to 5.1)	1.8 (1.2 to 2.9)	0.01	1.2 (0.8 to 1.9)	0.32
	Bisexual	3.7 (2.5 to 4.9)	4.7 (3.2 to 6.2)	2.0 (1.3 to 3.0)	0.00	1.3 (0.9 to 1.8)	0.15
	Unsure	1.6 (0.9 to 2.3)	6.5 (4.7 to 8.3)	1.0 (0.6 to 1.7)	0.85	1.5 (1.1 to 2.0)	0.01
Language other than English at home							
	No	2.0 (1.5 to 2.4)	3.6 (3.1 to 4.2)	Ref	Ref	Ref	Ref
	Yes	1.8 (1.3 to 2.4)	6.0 (5.0 to 6.9)	0.9 (0.6 to 1.3)	0.48	1.3 (1.04 to 1.71)	0.02
Perceived Tobacco Danger							
	No	4.1 (3.0 to 5.3)	9.6 (7.9 to 11.4)	Ref	Ref	Ref	Ref
	Yes	1.7 (1.4 to 2.1)	3.8 (3.3 to 4.3)	0.5 (0.3 to 0.6)	<.0001	0.4 (0.3 to 0.5)	<.0001

Abbreviations, CI: Confidence Interval; AOR: Adjusted Odds Ratio; NH: non-Hispanic.
[a]: Multinomial logistic regression model included current vaping CBD (no as the reference group) as the outcome variable with all variables in this Table as simultaneous regressors. Missing covariate data were managed with multiple imputation using 20 multiply-imputed data sets.

© 2023 Dai HD et al. *JAMA Network Open.*

eTable 2. Factors Correlated with Ever Vaping CBD among Ever E-cigarette Users

	Prevalence of Ever Vaping CBD		Ever Vaping CBD: Yes vs. No		Ever Vaping CBD: I don't know vs. No	
	Yes	Don't Know	AOR[a]	P-value[a]	AOR[a]	P-value[a]
	27.4 (25.2 to 29.7)	11.1 (9.5 to 12.6)				
Sex						
Male	25.6 (22.5 to 28.7)	10.7 (8.8 to 12.7)	Ref	Ref	Ref	Ref
Female	28.8 (25.7 to 32.0)	11.3 (9.3 to 13.2)	1.4 (1.1 to 1.8)	0.02	1.1 (0.8 to 1.4)	0.53
Grade						
Middle School	18.7 (14.4 to 23.0)	18.2 (13.8 to 22.5)	Ref	Ref	Ref	Ref
High School	29.3 (27.1 to 31.5)	9.3 (7.8 to 10.8)	1.5 (1.1 to 1.9)	0.01	0.6 (0.4 to 0.8)	0.003
Race / Ethnicity						
NH-white	27.0 (24.1 to 29.9)	9.1 (7.4 to 10.8)	Ref	Ref	Ref	Ref
NH-black	32.2 (27.7 to 36.8)	11.2 (7.6 to 14.8)	1.4 (1.0 to 2.0)	0.05	1.3 (0.8 to 2.0)	0.31
Hispanic	27.5 (23.8 to 31.3)	15.8 (12.8 to 18.8)	1.2 (0.9 to 1.6)	0.16	1.6 (1.1 to 2.3)	0.01
Others	23.4 (14.7 to 32.1)	8.9 (4.0 to 13.8)	0.9 (0.5 to 1.6)	0.77	0.8 (0.4 to 1.6)	0.50
Sexual Minority						
Heterosexual	25.1 (22.6 to 27.7)	10.6 (8.5 to 12.7)	Ref	Ref	Ref	Ref
Gay/Lesbian	30.4 (20.7 to 40.0)	11.2 (4.4 to 18.1)	1.1 (0.7 to 1.7)	0.60	1.0 (0.6 to 1.7)	0.93
Bisexual	29.3 (24.7 to 33.9)	12.2 (8.1 to 16.4)	1.2 (0.9 to 1.6)	0.28	1.1 (0.7 to 1.8)	0.71
Unsure	32.7 (24.4 to 41.1)	13.0 (6.5 to 19.6)	1.4 (0.9 to 2.0)	0.13	1.2 (0.6 to 2.5)	0.56
Language other than English at home						
No	26.5 (24 to 29.1)	9.4 (7.7 to 11.0)	Ref	Ref	Ref	Ref
Yes	25 (20.9 to 29.1)	15.8 (12 to 19.7)	1.1 (0.8 to 1.5)	0.63	1.3 (0.9 to 1.9)	0.20
Perceived Tobacco Danger						
No	34.1 (29.0 to 39.1)	10.4 (7.6 to 13.3)	Ref	Ref	Ref	Ref
Yes	25.0 (22.5 to 27.4)	11.2 (9.3 to 13.0)	0.7 (0.6 to 0.9)	0.02	1.0 (0.7 to 1.4)	0.93
Tried Vaping (years)						
<1	11.3 (8.2 to 14.4)	16.9 (13.3 to 20.5)	Ref	Ref	Ref	Ref

© 2023 Dai HD et al. *JAMA Network Open.*

	1	22.6 (19.1 to 26.0)	10.0 (7.4 to 12.6)	2.1 (1.4 to 3.0)	0.0002	0.7 (0.5 to 0.9)	0.02
	2-3	30.3 (26.6 to 34.1)	10.1 (7.7 to 12.6)	2.7 (1.9 to 4.0)	<.0001	0.8 (0.5 to 1.2)	0.28
	>3	41.9 (38.0 to 45.7)	9.7 (6.5 to 12.9)	4.1 (2.8 to 6.0)	<.0001	0.9 (0.6 to 1.4)	0.61
Ever use of other tobacco products							
	No	16.9 (13.9 to 19.8)	10.6 (8.9 to 12.3)	Ref	Ref	Ref	Ref
	Yes	37.5 (34.5 to 40.5)	11.5 (9.2 to 13.9)	2.7 (2.1 to 3.4)	<.0001	1.6 (1.2 to 2.1)	0.002

Abbreviations, CI: Confidence Interval; AOR: Adjusted Odds Ratio; NH: non-Hispanic.

[a]**:** Multinomial logistic regression model included current vaping CBD (no as the reference group) as the outcome variable with all variables in this Table as simultaneous regressors. Missing covariate data were managed with multiple imputation using 20 multiply-imputed data sets.

© 2023 Dai HD et al. *JAMA Network Open.*
