# Supplementary material for: Prevalence and Factors Associated With Vaping Cannabidiol Among US Adolescents
Source: JAMA Netw Open. 2023 Aug 16;6(8):e2329167. doi: 10.1001/jamanetworkopen.2023.29167 (PMC10433080; doi:10.1001/jamanetworkopen.2023.29167)
Supplement: Supplement 2. — Data Sharing Statement [file jamanetwopen-e2329167-s002.pdf]

Data Sharing Statement

Dai. Prevalence and Factors Associated With Vaping Cannabidiol Among US Adolescents. *JAMA Netw Open*. Published August 16, 2023. doi:10.1001/jamanetworkopen.2023.29167

Data
Data available: Yes
Data types: Deidentified participant data
How to access data: This is the secondary analysis of publicly available data, which can be accessed at https://www.cdc.gov/tobacco/data_statistics/surveys/nyts/index.htm.
When available: With publication

Supporting Documents
Document types: Other (please specify)
Additional Information: This is the secondary analysis of publicly available data, which can be accessed at https://www.cdc.gov/tobacco/data_statistics/surveys/nyts/index.htm.
How to access documents: Dataset and codebook
When available: With publication

Additional Information
Who can access the data: anyone
Types of analyses: This is the secondary analysis of publicly available data, which can be accessed at https://www.cdc.gov/tobacco/data_statistics/surveys/nyts/index.htm.
Mechanisms of data availability: https://www.cdc.gov/tobacco/data_statistics/surveys/nyts/index.htm.
